# The lungs of the finch: three-dimensional pulmonary anatomy of the zebra finch (*Taeniopygia castanotis*)

**DOI:** 10.1098/rstb.2023.0420

**Published:** 2025-02-27

**Authors:** Aracely Martinez, Raul E. Diaz Jr, Clinton A. Grand Pre, Brandon P. Hedrick, Emma R. Schachner

**Affiliations:** ^1^ Department of Cell Biology and Anatomy, Louisiana State University Health Sciences Center, New Orleans, LA 70112, USA; ^2^ Department of Biological Sciences, California State University Los Angeles, Los Angeles, CA 90032, USA; ^3^ Department of Anatomical Sciences, Renaissance School of Medicine, Stony Brook University, Stony Brook, NY 11794, USA; ^4^ Department of Biomedical Sciences, Cornell University College of Veterinary Medicine, Ithaca, NY 14853, USA; ^5^ Department of Physiological Sciences, University of Florida College of Veterinary Medicine, Gainesville, FL 32608, USA

**Keywords:** bronchi, segmentation, Aves, air sac, passerine, microCT

## Abstract

The avian respiratory system has been an area of biological interest for centuries, with zebra finches (*Taeniopygia castanotis*) emerging in recent decades as a primary avian model organism popularized across numerous disciplines. The pulmonary system of birds is unique in that air moves unidirectionally through the gas-exchanging lung, and previous works have suggested anatomical constraints within the bronchial network that may be coupled to the inspiratory valving mechanism in Aves. We used µCT-based segmented models to visualize and describe the morphology of the zebra finch lower respiratory system and to examine intra- and interspecific differences of the bronchial tree with the phylogenetically and ecologically different African grey parrot (*Psittacus erithacus*). Here, we show that zebra finches have highly variable lung and air sac morphology within individuals but generally do not diverge from the anatomical *bauplan* previously described for passerines. Additionally the parabronchi in the zebra finch lung are arranged into isolated segments between secondary bronchi, which has not been described and may be coupled with airflow patterns in this species. Both zebra finches and African grey parrots show constrained interostial distances and robust, caudally directed third ventrobronchi that may play an unexplored role in the unidirectional airflow patterns of birds.

This article is part of the theme issue ‘Biology of the avian respiratory system: development, evolutionary morphology, function and clinical considerations’.

## 1. Introduction

The zebra finch (*Taeniopygia castanotis*) is one of the most commonly studied avian model organisms in the fields of neuroscience [1,2], animal behaviour [3,4], reproductive biology [5], ecology [6], evolution [7,8], physiology [9–11] and genetics [12–15]. Previous studies have described the morphology of the upper respiratory system and the neural control required for certain aspects of sound production in songbirds [16–18]; however, the majority of existing research on the lower respiratory tract of songbirds has focused on the anatomy of the syrinx and its role in vocalization [8,19–23] with few studies examining the structure or function of the gas-exchanging lung and air sacs [24,25]. More recently, computed tomography (CT) and micro-computed tomography (µCT) have been used to assess skeletal and soft tissues *in situ* in birds and other closely related sauropsid taxa, which facilitate the creation of three-dimensional models of the avian respiratory system and its anatomical relationships to adjacent structures [26–29]. Passerines make up more than 50% of total extant bird species [30] and given the zebra finches’ interdisciplinary prevalence in biology, understanding how their respiratory systems relate to other birds and sauropsids is immensely valuable.

The avian respiratory system is composed of a unidirectionally ventilated, volume-constant gas-exchanging lung and a series of compliant, flexible ventilatory air sacs [31–35]. The lung parenchyma is immobilized and dorsally fixed within the coelomic cavity by a series of forked vertebral ribs and a horizontal septum along the ventral surface of the lung [33,36]. The primary bronchus gives off three distinct groups of secondary bronchi that are generally named for the surface from which they emerge and their position within the lung (i.e. ventro-, dorso-, laterobronchi) [32]. The secondary bronchi ramify into numerous tertiary branches called parabronchi that are arranged with gas-exchanging tissue packed densely in between them [37,38]. The parabronchial network is subdivided into paleopulmo and neopulmo. The paleopulmo is present in all birds and consists of parabronchi arranged in parallel where gas-exchange occurs as the air moves caudo-cranially from the dorsobronchi to the ventrobronchi [39]. The neopulmo is situated laterally in the lung and unlike the paleopulmo, it is composed of highly irregular and interconnected parabronchi that are variable in length. The neopulmo varies across Aves with respect to the percentage of lung volume it occupies. In species with a well-developed neopulmo, like songbirds, the neopulmo may occupy 10−20% of the parabronchial mass [32]. These species also have caudal parabronchi, which often converge to form saccobronchi that provide indirect connections between the secondary bronchi and caudal air sacs (i.e. caudal thoracic, abdominal) [40]. This complex tubular structure allows the parenchyma to remain patent and facilitate the movement of air in a caudal-to-cranial direction through a large surface area of gas-exchanging tissue [35]. At the margins of the lung, some of the secondary bronchi expand into air sacs that can be divided into cranial and caudal groups [31,37]. The cranial group of air sacs includes the cervical, interclavicular and cranial thoracic air sacs and the caudal group is composed of the abdominal and caudal thoracic air sacs [31,32,41]. Together, the immobilized lung parenchyma and flexible air sacs allow for a gas-exchanging organ that is decoupled from its ventilator [36,42].

Early in the last century, numerous studies aimed to explain the aerodynamic properties that are prerequisites for unidirectional airflow through the airways of the avian lung [43–45]. Experimental work has demonstrated that the shape of the primary bronchus and the ostia of the secondary bronchi produce direction-dependent resistance that facilitates unidirectional flow [35,46], which has been validated by simplified computational and fluid dynamic modelling [47–51]. Recent studies have found that unidirectional airflow also occurs in the lungs of crocodilians [52,53], varanids [54,55] and the green iguana [56]—taxa that all lack many features typically associated with the avian respiratory system (e.g. an immobilized lung, fully decoupled air sacs) and bronchial trees that vary substantially from the avian *bauplan*. These studies support the hypothesis that unidirectional flow predates flight and is likely ancestral for both archosaurs and perhaps even tetrapods [57,58]; however, substantial questions remain regarding which components of the bronchial tree are necessary to maintain unidirectional flow across sauropsids or even within Aves. For example, an inspiratory valve has been proposed to be the mechanism by which airflow bypasses a set of the secondary airways (the ventrobronchi) during the inhalatory phase of ventilation. The shape and arrangement of the secondary airways have been identified as key features in the bronchial tree of archosaurs that are proposed as critical to the inspiratory valve and provide potential homologies with other taxa [26,27]. However, comparative quantitative interspecific analyses between individual avian taxa have yet to be completed, and these studies will facilitate addressing questions regarding the degree to which the inspiratory valve is constrained across Aves and other structures associated with maintaining unidirectional airflow patterns.

Here, we use µCT imaging data and three-dimensional anatomical modelling to visualize and describe the intraspecific variation in morphology of the zebra finch lower respiratory system. Additionally, we compare the zebra finch lower respiratory system interspecifically with that of the African grey parrot (*Psittacus erithacus*), a popular pet species that is also of interest due to their behaviour, intelligence and ability to mimic human speech [59–61], to test predictions about shared aspects of the bronchial tree that are hypothesized to be coupled to the inspiratory valve. Specifically, we expect that the avian bronchial tree will exhibit less anatomical variance in close proximity to the ventrobronchi due to the constraint of the inspiratory aerodynamic valve. By examining the small (10−15.2 g) flap-bounding passeriform and comparing it to the medium sized (301–353 g) continuous flapping psittaciform [62–64], this work seeks to test the hypothesis that despite divergent phylogenetic positions, size and behavioural differences, the bronchial tree should be functionally constrained due to the nature of shared unidirectional airflow patterns.

## 2. Methods

### (a) Specimens

Micro-computed tomography scans were obtained from adult zebra finch specimens (*n* = 5) at the University of Southern California Molecular Imaging Center on a Nikon XT H 225kV µCT scanner (table 1). For ease of identifying individuals, specimens were named for gods in Aztec mythology (table 1). Naturally deceased and frozen specimens were donated to the Diaz Lab from a local Los Angeles pet store and allowed to thaw prior to scanning. Four specimens were intubated with a 20 gauge catheter, three of which were manually inflated with a syringe to their maximum inspiratory capacity (MIC) until resistance was felt. The unintubated and failed intubation specimens (*n* = 2) were imaged at their natural end tidal volume (NETV). The zebra finch imaging data will be available via MorphoSource in DICOM format with acceptance of the article. Comparative µCT data for adult African grey parrots (*n* = 9) were obtained from published datasets available via Data Dryad [65].

**Table 1.** *Taeniopyga castanotis* specimen and scan data. MIC, maximum inspiratory capacity; NETV, natural end tidal volume.

specimen name	specimen number	sex	mass (g)	status at scan	scan resolution (μm)
Quetzalcoatl	ERS2022−027	M	11.49	no intubation, NETV	38.954
Xolotl	ERS2022−028	M	11.29	intubated and inflated to MIC	48.130
Tezcatlipoca	ERS2022−029	F	12.46	intubated and inflated to MIC	44.750
Mixcoatl	ERS2022−030	M	12.29	intubated and inflated to MIC	55.886
Xipe Totec	ERS2022−031	M	11.94	Intubation failed, NETV	44.967

### (b) Anatomical modelling

Three-dimensional surface models of the gas-exchanging lung and air sacs, bronchial tree, parabronchial network and skeleton for the zebra finch specimens were segmented in AvizoLite 2020.2 (Thermo Fisher Scientific) following the methods used for extant archosaur lungs [26,28]. Using the thresholding tool, the values for negative air space were selected in all slices to build a rough model of the full volume of the respiratory system for each specimen. A Wacom Intuos Pro pen tablet was used along the boundaries between the lung, air sacs, diverticula and bronchial network to separate each structure. Manual segmentation and interpolation techniques were used when previous thresholding was insufficient.

### (c) Bronchial terminology

The anatomical terminology for the bronchial structures in the avian lung has been widely disputed in the literature (see table 1 in Lawson *et al*. [26] for a historical comparison of the terminology). Unidirectional airflow was originally assumed to be restricted to birds, but studies of airflow patterns in crocodilian, varanid and iguana lungs in recent decades has shown that this trait is likely ancestral for Sauropsida [52,54,56,66] and at the very least, crocodylian lung morphology should be factored into hypotheses of bronchial homology for archosaurs [27,53,67]. The terminology used in the present study follows that which was utilized by Duncker [32] and Schachner *et al*. [27], which clusters the airways into distinct functional units that can be compared interspecifically across orders of birds for clarity of communication. The secondary bronchi are termed based on both their branching location from the intrapulmonary primary bronchus and their position within the lung (i.e. ventro-, dorso-, laterobronchi). Most birds typically have four ventrobronchi (also called medioventral secondary bronchi or occasionally craniomedial secondary bronchi), seven or more dorsobronchi (also called medio- or laterodorsal secondary bronchi, or occasionally craniodorsal secondary bronchi), and a series of highly variable laterobronchi that branch off of the ventral and lateral aspects of the mid and caudal length of the intrapulmonary primary bronchus (also called caudoventral or lateroventral secondary bronchi). (See figure 2*a* for a clear diagram.) Using more direct terms allows for clarity of communication of the secondary bronchi and retains ‘functional clusters’ for comparative analysis between diverse avian taxa. In addition, these terms allow for hypotheses of homology across Archosauria (i.e. with crocodylians).

### (d) Quantitative measures and analysis

Air sac volumes were obtained from the three-dimensional segmented models in AvizoLite 2020.2 for all five specimens; however, inflation pressures were not collected prior to scanning. Total parabronchial volumes for the paleo- and neopulmo were obtained for Quetzalcoatl and Tezcatlipoca. In these two specimens, parabronchi in each paleopulmonic segment were quantified by counting the total number of terminal branches arising from the walls of the connected ventro- and dorsobronchi that fuse. This method was chosen rather than counting the number of parabronchial ostia arising from the walls of the secondary bronchi because during development, parabronchi often bifurcate as they grow distally towards other parabronchi and fuse to form an interconnected network [68].

To make intraspecific quantitative comparisons between the zebra finches, and then compare their bronchial trees with those of the parrot specimens, multiple measures were collected from the µCT data for each bronchus in OsiriX MD [69]. Measurements of the parrot bronchial trees were obtained from Lawson *et al.* [26]. Airway measurements are based on the hypothesized homologous structures established by previous studies [26,27,53,67] and respiratory development [70,71]. The closed polygon and length tools in the Multiplanar Reconstruction (MPR) Viewer were used to collect the following metrics: (i) cross-sectional areas of the primary bronchus just proximal to the ostium of each secondary bronchus; (ii) cross-sectional areas of secondary bronchial ostia; (iii) distance from the carina to V1; and (iv) inter-ostial distances between each ventro- and dorsobronchus. For ease of comparison, parrot specimens with the most common ventrobronchial phenotype (V1–V5) were used. Parabronchial diameters were collected in OsiriX MD for all paleopulmonic segments and the neopulmo; measurements were taken three times and averaged for each zebra finch specimen. Residuals were not used as initial regression analysis against mass in all specimens showed no significant allometric relationship in the data. The standard error of the mean (s.e.m.) was calculated to assess variability within each group of metrics collected from the primary and secondary bronchi and interostial distances. Data visualization and s.e.m. calculations were performed using the basic statistics and plotrix (v3.8.4) packages in R [72,73]. Differences between primary and secondary bronchial cross-sectional areas and the interostial distances for the secondary bronchi in both species were graphically expressed in boxplots and the s.e.m. values for each metric were represented using the ggplot2 package (v3.5.1) [74].

## 3. Results

### (a) Lung and bronchial tree morphology

**Figure 1.**
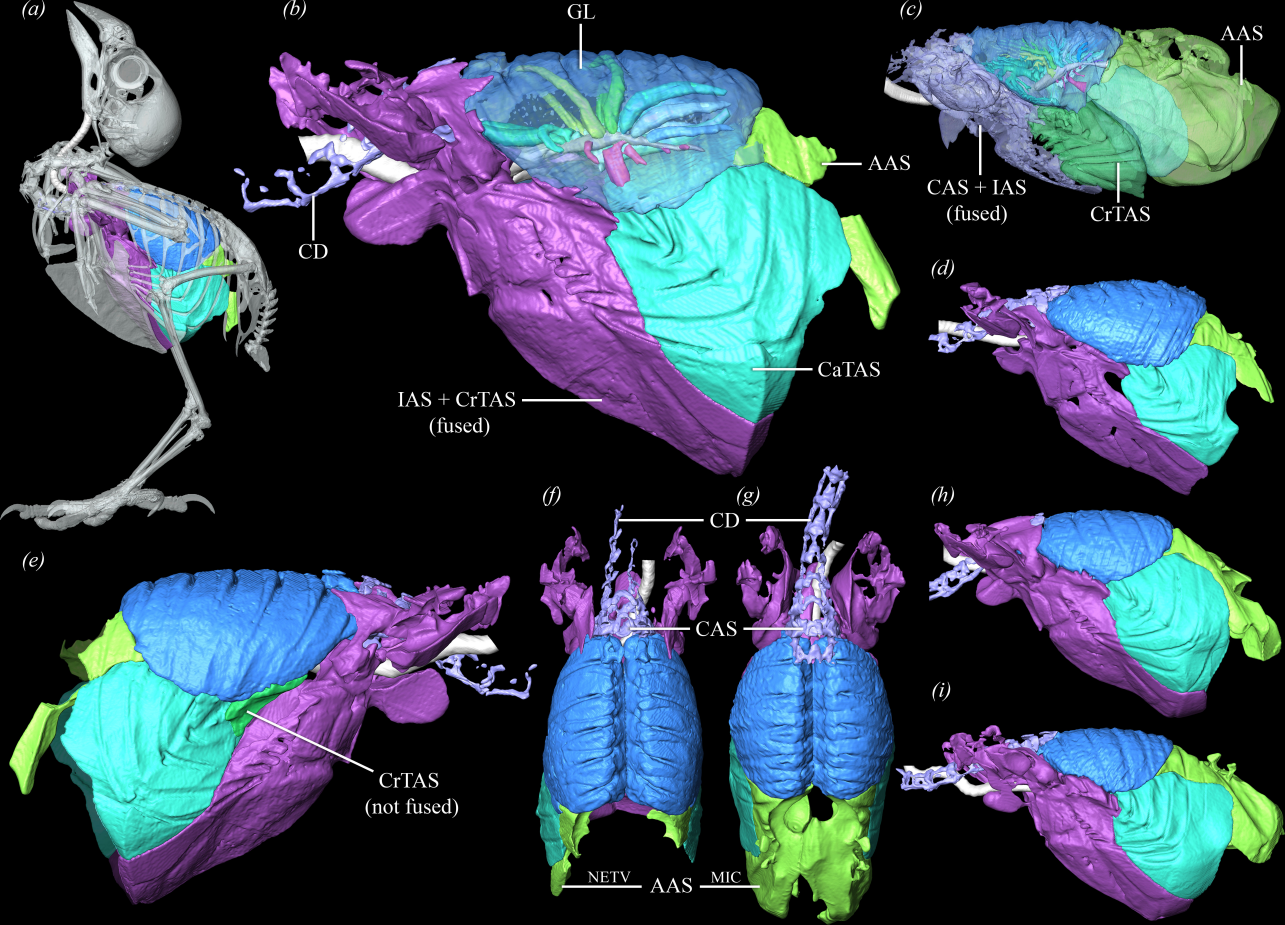
Left lateral views of the lower respiratory tract of *T. castanotis*. (*a*) Lungs and air sacs with intact skeleton. (*b,d,h,i*) Left lateral views of different specimens. (*e*) Right lateral view of respiratory tract demonstrating unfused CrTAS and IAS. Specimen in (*a,b,e*) was imaged at NETV. Dorsal views of lungs and air sacs at NETV (*f*) and MIC (*g*). (*c*) Left lateral view of lower respiratory tract of *P. erithacus* [26]. Abbreviations: AAS, abdominal air sac; CAS, cervical air sac; CaTAS, caudal thoracic air sac; CD, cervical diverticula; CrTAS, cranial thoracic air sac; GL, gas-exchanging lung; IAS, interclavicular air sac; MIC, maximum inspiratory capacity; NETV, natural end tidal volume.

**Figure 2.**
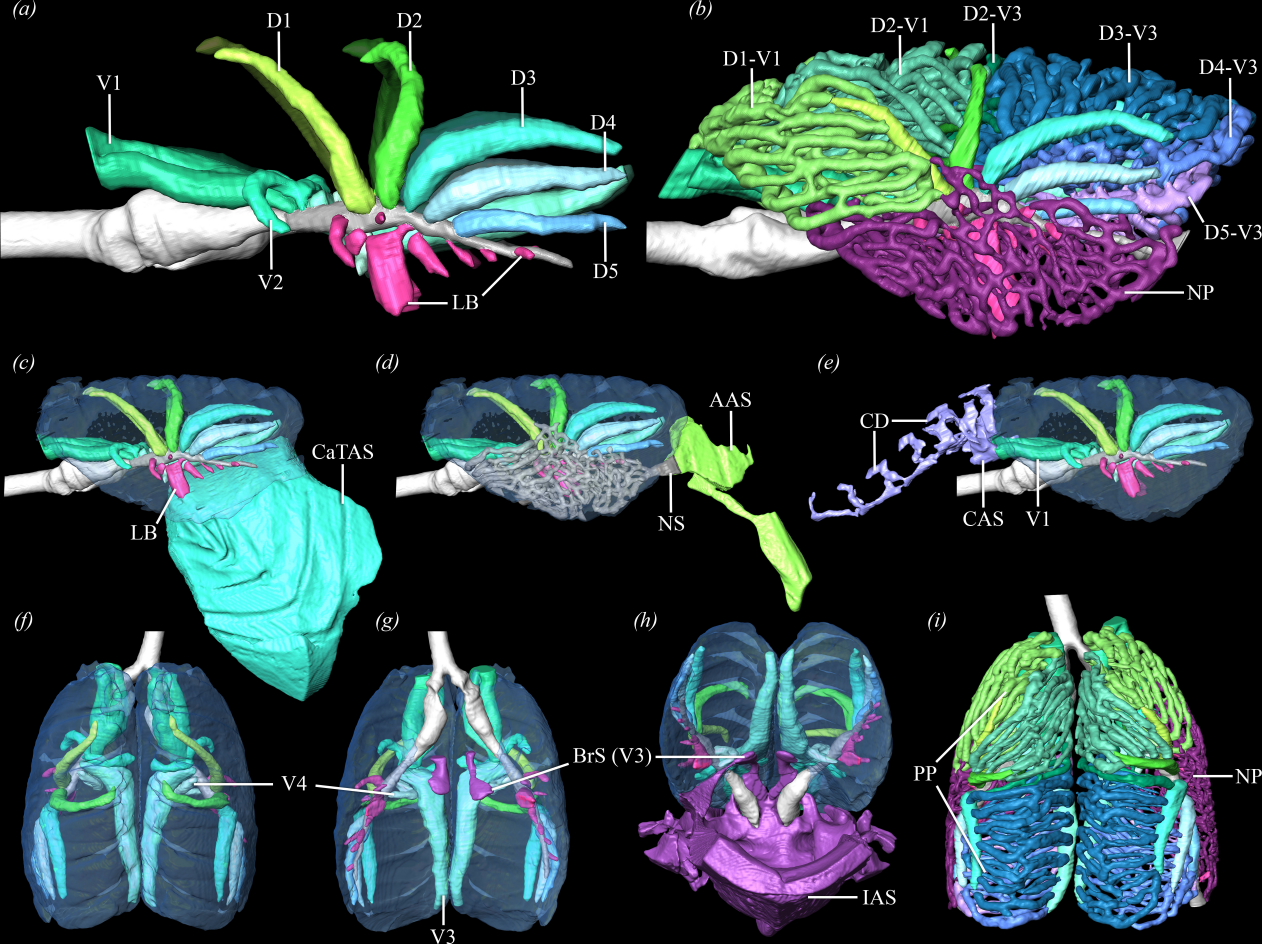
Left lateral views of the bronchial tree in *T. castanotis* with (*a*) primary and secondary bronchi (Quetzalcoatl) and (*b*) paleo- and neopulmo (Tezcatlipoca). (*c–e*) Left lateral views of Quetzalcoatl’s bronchial connections to air sacs. Dorsal (*f*) and ventral (*g*) views of the bronchial tree; caudoventral view (*h*) with BrS on ventral surface of V3 connecting to IAS; (*i*) dorsal view of paleo- and neopulmo. Abbreviations: AAS, abdominal air sac; BrS, bronchial stem; CAS, cervical air sac; CaTAS, caudal thoracic air sac; CD, cervical diverticula; IAS, interclavicular air sac; LB, laterobronchus/i; NP, neopulmo; NS, neopulmonic saccobronchus; PP, paleopulmo.

The gas-exchanging lung in the zebra finch spans from the first thoracic vertebra (T1) to the cranial edge of the synsacrum (figure 1*a*). The primary bronchus enters the lung and immediately gives off four ventrobronchi (V1−4) which arise from its dorsomedial surface (figure 2*f*,*g*). The only exception is V2, which is small and laterally displaced (figure 2*a*). All zebra finches in this study have five dorsobronchi (D1−5) that form the superficial surface of the dorsolateral aspect of the gas-exchanging lung. There are 7−13 laterobronchi that emerge from the primary bronchus and contribute to the parabronchi making up the neopulmo.

The primary bronchus in the zebra finch is widest between V4 and D1 and following the branching of D1 it decreases in diameter to the size of a parabronchus (figure 2*a*). The primary bronchus typically joins with adjacent parabronchi to form a funnel-shaped saccobronchus that serves as a robust ostium into the abdominal air sac. This is demonstrated very clearly in Quetzalcoatl (left lung), Xolotl (left lung) and Mixcoatl (figure 2*d*). Tezcatlipoca has primary bronchi that dilate and receive some neopulmonic parabronchi to form an ostium connecting to the abdominal air sac (figure 2*b*,*i*). Three specimens have a primary bronchus that loses any connection, direct or indirect, to the abdominal air sac completely. Xipe Totec and Quetzalcoatl (right lung) reduce the diameter of their primary bronchi and come to a blind end just proximal to the caudal margin of the gas-exchanging lung. Xolotl (right lung) has a primary bronchus that loses its connection to the abdominal air sac by curving ventrally and cranially to become a part of the neopulmo.

**Figure 3.**
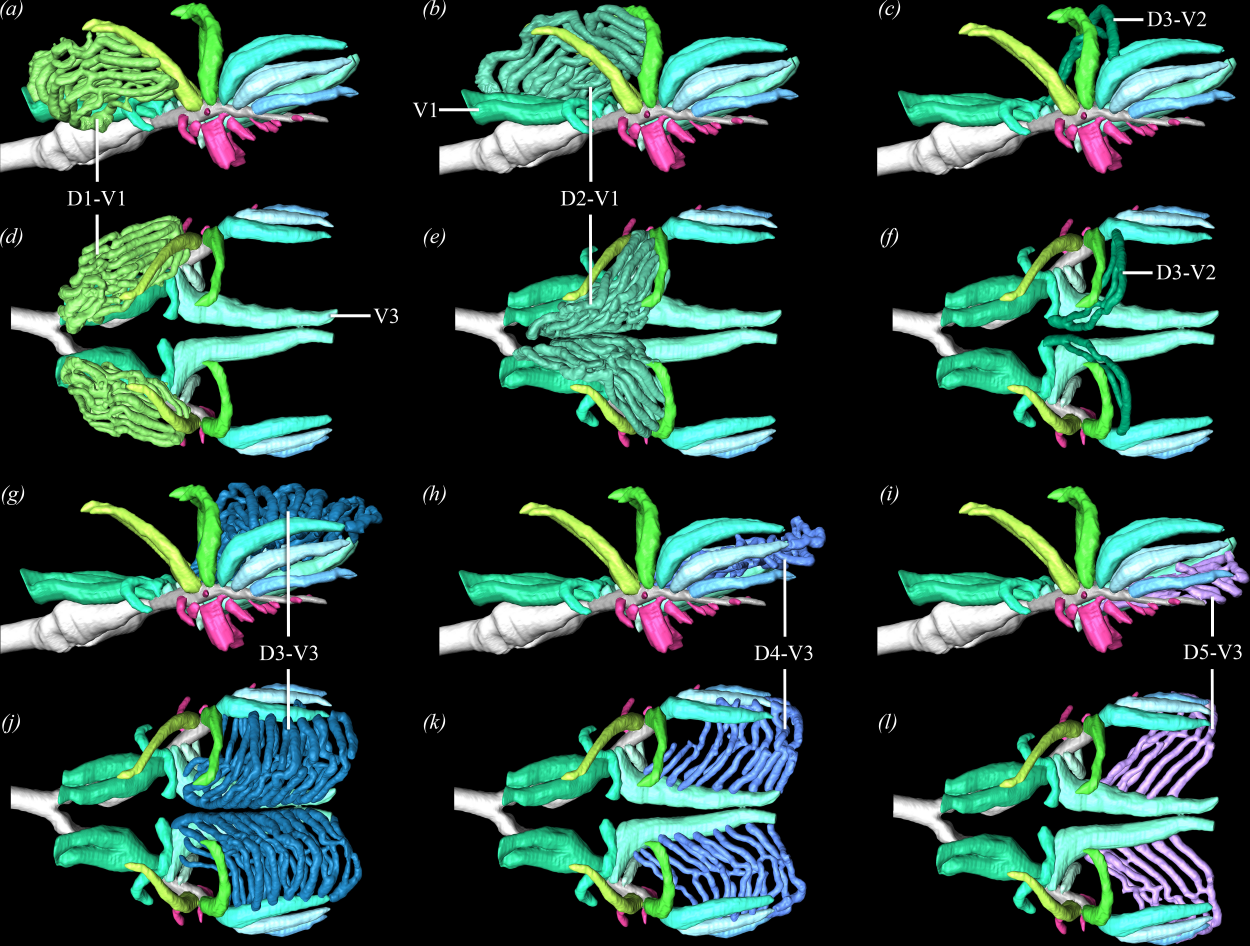
Left lateral views (*a–c,g–i*) and dorsal (*d–f, j–l*) of the paleopulmonic segments of Quetzalcoatl. Abbreviations: D, dorsobronchus; V, ventrobronchus.

The paleopulmo is composed of parabronchi positioned between the dorsobronchi and ventrobronchi that are arranged in parallel (figure 3), ranging between 315 and 317 total branches (left: 153−159; right: 153−162), making up 76−82% of the total parabronchial volume (electronic supplementary material, table S4). The present use of µCT data and segmentation allows the paleopulmo to be separated into distinct sections that connect one dorsobronchus to one ventrobronchus with very little cross-linking with adjacent parabronchi. The first dorsobronchus (D1) courses cranially, decreasing in diameter until it is the size of a parabronchus—approximately 220 µm in diameter (averaged for the paleopulmo; figure 2*a*). Parabronchi (150–270 µm in diameter) connect the cranial surface of D1 to the dorsal and lateral surfaces of the two main branches of the first ventrobronchus (V1) proximal to their formation of the cervical air sac ostium (figure 3*a*,*d*). These 29−34 parabronchi make up the craniolateral aspect of the paleopulmo, and the ventrolateral branches often contribute to the cranial portion of the neopulmo (figure 2*b*). In Tezcatlipoca, an additional segment exists between D1 and V1, which are connected by two to three parabronchi just caudal to the medial portion of the neopulmo.

The second dorsobronchus (D2) curves medially and slightly cranially, narrowing to about 210 µm in diameter (figure 2*a*). Between the cranial and ventral surfaces of D2 and the medial branch of V1, 25−30 parabronchi (197–263 µm in diameter) connect to make up the craniomedial surface of the paleopulmo (figure 3*b*,*e*). There are three to five small parabronchi connecting the proximal ventral surface of D2 with the medial branch of V2. These are the only parabronchi that connect to the medial branch of V2 that do not contribute to the neopulmo, although they occasionally have a connection to the adjacent dorsal parabronchi, linking them to the D2–V1 segment.

The third dorsobronchus (D3) has parabronchial connections to V2 and V3, making up the middle portion of the paleopulmo. Both D3 and the medial branch of V2 have a single parabronchus emerging from their dorsal surfaces that courses cranially, branches into four to six parallel parabronchi that merge and lack any connections to adjacent segments (figure 3*c*,*f*). The largest paleopulmonic segment by volume (8.29−12.74 mm^3^) and in number of parabronchi (43–49 branches, ranging 170−303 µm in diameter) is found between the medial and dorsal surfaces of D3 and the dorsal surface of V3 (figure 3*g*,*j*; electronic supplementary material, table S4). The ventral, cranialmost of these branches have connections to adjacent parabronchi in the D2–V1 segment of the paleopulmo (figure 2*i*). Distally, both D3 and V3 narrow and bifurcate into two parabronchi that communicate with one another. V3 is a large tube-shaped bronchus that, unlike V1 and V2, after emerging from the primary bronchus instead of making a hair-pin turn cranially, it immediately turns caudally and runs the length of the medioventral aspect of the gas-exchanging lung (figure 2*f*,*g*). Bilaterally, V3 gives off a bronchial stem emerging from its ventral surface that expands cranially and caudally into the impar interclavicular and cranial thoracic air sacs, respectively (figure 2*g*,*h*).

There are 24−38 parabronchi (167–233 µm in diameter) connecting medial and dorsal surfaces of the fourth dorsobronchus (D4) to the medial surface of V3, located just ventral to the D3–V3 segments (figures 2b,3h,k). The caudalmost portion of the paleopulmo is made up of 12−17 parabronchi (177–256 µm in diameter) connecting the medial and ventral surfaces of the fifth dorsobronchus (D5) to the ventromedial surface of V3 and are situated just ventral to the D4–V3 segments. The parabronchi from D4 and D5 occasionally have connections with adjacent segments (figure 3*i*,*l*). The fourth ventrobronchus (V4) is small and, like V3, branches off in a caudal direction, but immediately wraps laterally and ventrally to form a C-shaped tube around the pulmonary artery and primary bronchus (figure 2*f*,*g*).

The neopulmo in the zebra finch occupies 17−23% of the total parabronchial volume and is made up of numerous irregular and interconnected parabronchi ranging from 120 to 210 µm in diameter that emerge from V2, V3, V4, multiple laterobronchi and the lateral surfaces of all five dorsobronchi (figure 2*b*). V1 has no direct connections to the neopulmo, but ventrolateral branches in the D1–V1 segment of the paleopulmo may contribute to the cranial portion of the neopulmo. The lateral branch of V2 curves ventrally and contributes solely to the neopulmo. Similarly, V4 only contributes to the neopulmo, with no connections to any paleopulmonic parabronchi. V3 is the only ventrobronchus in the zebra finch that has no connections to the neopulmo. All five dorsobronchi have branches emerging from their lateral surfaces that become the dorsal and caudal regions of the neopulmo; this causes the dorsobronchi to be displaced slightly medially, away from the lateral lung surface. Caudally, three to five neopulmonic parabronchi converge to form a saccobronchus that generates a robust opening into the abdominal air sac, secondary to the primary bronchus (figure 2*d*). Cranioventral neopulmonic parabronchi often create smaller ostial connections to the cranial thoracic air sacs.

### (b) Air sacs and diverticula

There is considerable variation in the lung-sac connections in the zebra finch. The cervical air sac originates at the cranial margin of the lung where two parallel branches of V1 converge into an ostium. The cervical diverticula extend as far cranially as the fifth cervical vertebra and often fuse along the midline within the neural canal to form the supramedullary diverticula. The interclavicular and cranial thoracic air sacs arise from a bronchial stem on the ventral surface of V3 (figure 2*g*,*h*) and are typically fused bilaterally in the zebra finch, their combined volume ranging from 200.52 to 668.78 mm^3^ (figure 1*b*,*d*,*h*,*i*). Two specimens (Quetzalcoatl and Xipe Totec) have interclavicular air sacs that lack fusion with the right cranial thoracic air sacs (4.80−9 mm^3^) but remain fused with the left cranial thoracic air sacs (figure 1*e*; electronic supplementary material, table S1). The cranial thoracic air sacs, regardless of fusion, also connect to neopulmonic parabronchi along the lateroventral surface of the gas-exchanging lung where they create multiple small ostia that communicate with these air sacs. A single large laterobronchus opens into the caudal thoracic air sacs, which are the largest of the caudal group of air sacs in the zebra finch, and range in volume from 112.90 mm^3^ (NETV) to 377.17 mm^3^ (MIC) for individual air sacs (figures 1*b* and 2*c*). The abdominal air sacs are smaller than the caudal thoracic sacs and range in volume from 5.46 mm^3^ (NETV) to 153.94 mm^3^ (MIC) (electronic supplementary material, table S1). The abdominal air sacs often retain connections to the primary bronchus via its fusion with caudal neopulmonic parabronchi that form a large saccobronchus. The primary bronchi may also lose any direct or indirect connection visible via µCT to the abdominal air sac by curving dorsally and cranially to become a parabronchus in the paleopulmo, or by coming to a blind end.

### (c) Pulmonary metrics

**Figure 4.**
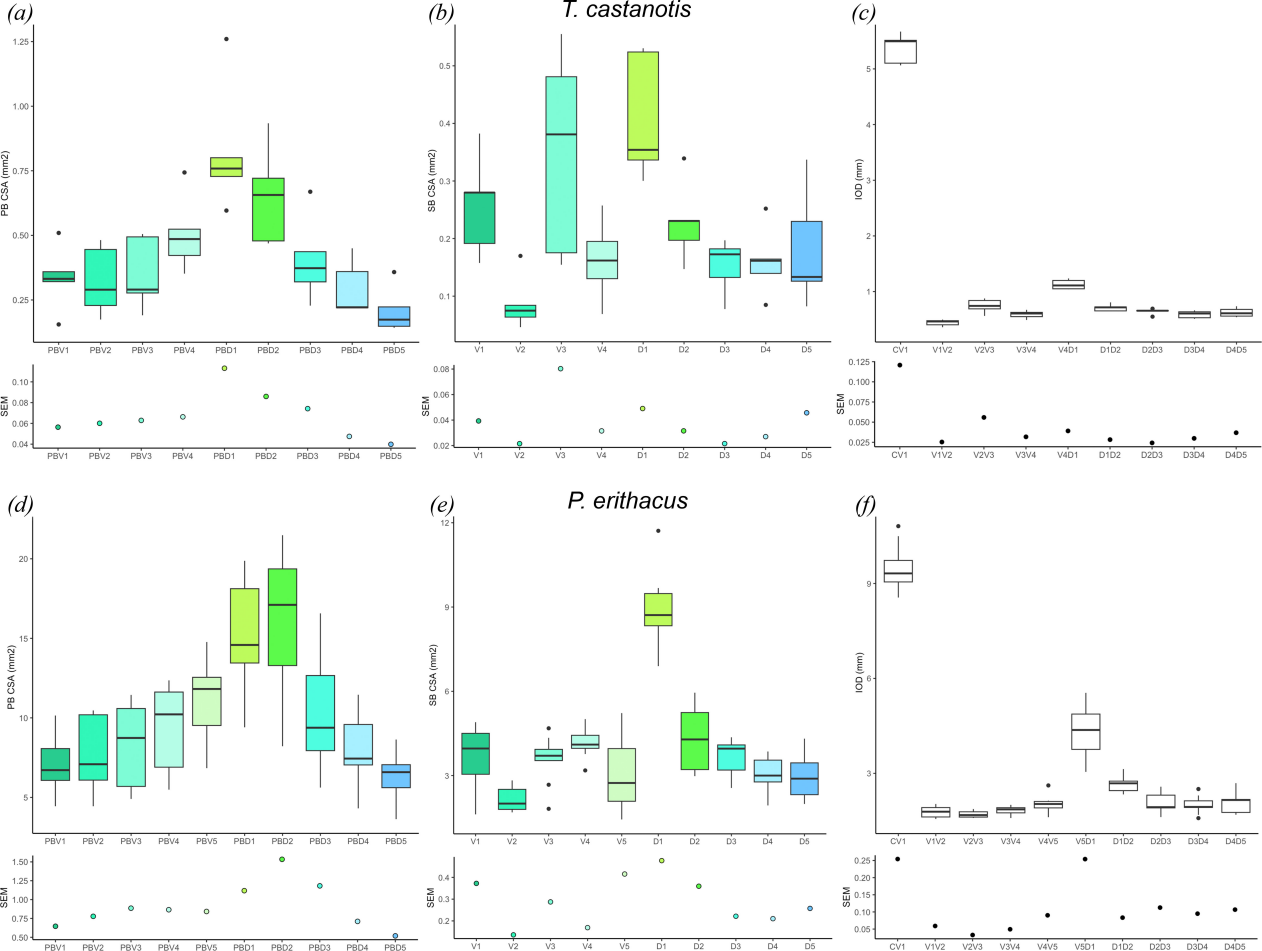
Plots of standard error of the mean for *T. castanotis* (*a–c*) and *P. erithacus* (*d–f*). (*a,d*) Cross-sectional areas of primary bronchi. (*b,e*) Cross-sectional areas of secondary bronchi. (*c,f*) Interostial distances. Colours correspond to secondary bronchi in figures 2 and 3. Abbreviations: CSA, cross-sectional area; IOD, interostial distance; PB, primary bronchi; SB, secondary bronchi; SEM, standard error of the mean. Abbreviations: C, carina; D, dorsobronchus; PB, primary bronchus; V, ventrobronchus.

Metrics collected from the bronchial tree of the zebra finch indicate that the primary bronchus progressively expands to its largest size at the level of D1 (0.59−1.26 mm^2^) (figure 4*a*), and then gradually constricts to approximately the size of a parabronchus (150–220 µm in diameter). Comparison of mean cross-sectional areas of the secondary bronchial ostia demonstrate that the V2 ostia are the smallest of the ventrobronchi group, and the dorsobronchial ostia gradually decrease in size from D1 (0.30−0.53 mm^2^) to D5 (0.08−0.33 mm^2^) (figure 4*b*). The cross-sectional areas of V3 (0.15−0.55 mm^2^, s.e.m. = 0.08 mm^2^) and D1 (0.30−0.53 mm^2^, s.e.m. = 0.049 mm^2^) are both the largest and the most variable in zebra finches (figure 4*b*). The least variable metrics were the primary bronchus cross-sectional area at V1 (0.15−0.50 mm^2^, s.e.m. = 0.056 mm^2^), D4 (0.21−0.44 mm^2^, s.e.m. = 0.047 mm^2^) and D5 (0.14−0.35 mm^2^, s.e.m. = 0.039 mm^2^) and the cross-sectional area of V2 (0.04−0.16 mm^2^, s.e.m. = 0.021 mm^2^), D3 (0.07−0.19 mm^2^, s.e.m. = 0.021 mm^2^) and D4 (0.08−0.25 mm^2^, s.e.m. = 0.026 mm^2^) (electronic supplementary material, table S2). The interostial distances in the zebra finch were tightly constrained, and the least variable from V1 to V2 (0.36−0.49 mm, s.e.m. = 0.025 mm), and D1 to D4 (0.51−0.8 mm, s.e.m. = 0.02 mm) (figure 4*c*).

## 4. Discussion

### (a) Morphology of the air sacs and diverticula

The definitive number and morphology of avian air sacs vary due to fusion of paired primordial air sacs during development in various species, and differences in the extent of air sacs within and beyond the coelomic cavity [32,40,71,75]. Typically, there are nine air sacs extending from the gas-exchanging lung: a single fused interclavicular sac, paired cervical, cranial and caudal thoracic, and abdominal air sacs [31,32]. The number of air sacs is known to vary from as few as five air sacs in hummingbirds (*Colibri coruscans*) that fuse their cervical, interclavicular and cranial thoracic air sacs, to as many as 14 air sacs in Ciconiiformes that retain paired medial and lateral clavicular sacs and may further divide the caudal thoracic air sacs to form an additional pair [31,40,75]. The most common fusion is that of the paired medial and lateral clavicular air sacs, which form a singular interclavicular air sac in the cranioventral region of the coelomic cavity [70,71]. In the zebra finch there are seven to eight air sacs [32]. As in pheasants, pigeons and penguins, zebra finches exhibit cranial thoracic air sacs that are small in comparison to the caudal thoracic air sacs (electronic supplementary material, table S1), and as described in other passerines, the interclavicular and cranial thoracic air sacs are often fused [32,75]. Zebra finches are intraspecifically variable here, demonstrating this fusion unilaterally in some individuals and bilaterally in others (figure 1*b*,*e*). In individuals with unilateral fusion (Quetzalcoatl and Xipe Totec), the cranial thoracic air sac retains multiple small connections to neopulmonic parabronchi along its dorsal surface. In Quetzalcoatl, these are the only connections to the unpaired cranial thoracic air sac as it has lost the connection to the V3 bronchial stem. Xipe Totec has bilateral connections from the cranial thoracic air sac to the bronchial stem and neopulmonic parabronchi bilaterally. Rather than a distinct bronchial stem, parrots have two direct ostia on the ventral surface of V3 that open into the interclavicular air sac, as well as a few small, indirect ostia originating from the adjacent parabronchial network [26].

The cervical air sacs are small, paired structures in the cranialmost aspect of the coelomic cavity that range in size from large and pronounced (e.g. penguins, buzzards) to very small (e.g. storks, herons, passerines) [40,76]. In the zebra finch, these air sacs are relatively small and do not fuse with the interclavicular air sac (figure 1*f*–g, electronic supplementary material, table S1), as seen in African grey parrots and hummingbirds [26,75].

Passerines reduce the volume of their abdominal air sacs and maintain connections to caudal parabronchi that fuse to form a saccobronchus distinct from the substantial direct connection to the primary bronchus seen in other avian taxa [32,75]. The primary bronchus and abdominal air sac morphology of the zebra finch varies from completely lacking a connection to merging with adjacent neopulmonal parabronchi to form a robust saccobronchial connection (figure 2*d*). This differs from that of the African grey parrot, ducks, pigeons, carrion crows and ostriches, which consistently demonstrate the more commonly described avian anatomy of a primary bronchus that dilates into an ostium connecting the abdominal air sac at the caudal margin of the gas-exchanging lung (figure 1*c*) [26,27,32,40]. However, most other birds also exhibit varying degrees of numerous indirect parabronchial connections to the abdominal air sacs (i.e. saccobronchi) that are lateral to and smaller than the primary bronchial ostium (e.g. parrots, ducks, cranes, chickens) [26,32].

The caudal thoracic air sacs in the zebra finch follow the general avian *bauplan* as they originate from a large laterobronchus and are situated ventrolaterally to the abdominal air sac, and as described for passerines (e.g. carrion crow, house sparrow, budgerigar), hummingbirds, penguins, petrels and herons, it is much larger in comparison to the abdominal air sac (electronic supplementary material, table S1) [31,75–77]. Of note, in zebra finches, the ostium of the laterobronchus on the primary bronchus is positioned directly opposite from the ostia for D3 and D4 which may function to draw air from the caudal thoracic air sac and into the paleopulmonic parabronchi where gas exchange occurs during exhalation (figure 2*a*). This alignment of the laterobronchus and third dorsobronchus *and* the reduction in size and connections to the bronchial tree of the abdominal air sac may be a result of the caudal thoracic air sac taking over as the primary caudal air reservoir for the inhalatory portion of the ventilation cycle. The parrots have caudal thoracic air sacs that are smaller than the abdominal air sacs, much like penguins, ducks, swans and the common cuckoo [31,32,40,76]. The large laterobronchial ostium is variable in the African grey parrot and may be oriented towards the cranial and middle dorsobronchi, or slightly caudally as in the zebra finch [26].

Zebra finches exhibit cervical diverticula that fuse with the cervical air sac, but often retain their proximal connections to the craniomedial margin of the gas-exchanging lung where paleopulmonic parabronchi converge and give rise to these diverticula (figure 1*f*,*g*). Developmental studies are required to determine when the fusion between the cervical air sac and diverticula occur and if these diverticula are derived from the cervical sac and ventrobronchi, or solely from the parabronchi and fuse with the cervical sac later in ontogeny. The interclavicular air sac in the zebra finches and African grey parrots gives rise to axillary diverticula that surround the pectoral girdle, but are not nearly as extensive as these diverticula in other birds, which can extend into the pectoral musculature (e.g. hawks, pelicans) [29] and even subcutaneously (e.g. pelicans, hornbills, albatross, flamingoes) [31]. Zebra finches and African grey parrots also produce a single midline diverticular process from the interclavicular air sac that occupies the space just dorsal to the sternum and may act as some sort of functional cushion for the trachea as it enters the coelomic cavity. African grey parrots also have diverticula emerging from the abdominal air sac that course laterally towards the femoral head and dorsally to the renal fossa of the synsacrum [26].

### (b) The parabronchi and ventrobronchus 3

In zebra finches, the paleopulmo and neopulmo make up 76−82% and 18−24% of the total parabronchial volume respectively, which is consistent with findings for passerines [32,37,40]. The parabronchi in the paleopulmo are arranged in parallel with few interconnections and have diameters that are large relative to body size (181–272 µm), allowing them to be separated into distinct segments (figures 2 and 3; electronic supplementary material, tables S3, S4). Connections between adjacent segments are few and typically arise in the caudal paleopulmo and laterally where neopulmo and paleopulmo communicate. In African grey parrots, the parabronchi are substantially smaller in diameter making it challenging to determine if distinct isolated segments are present as in the zebra finch, or if the paleopulmo is as highly interconnected in a similar organized manner.

Visualizing distinct parabronchial segments in zebra finches through anatomical modelling demonstrates a level of functional organization in the avian gas-exchanging lung that has previously been unavailable for three-dimensional analysis due to the small size of the parabronchial lumen. It is interesting that the parabronchial lumen are so large in these taxa, considering that their flap-bounding flight style requires flight costs greater than 25 x their basal metabolic rate [63,78]; however, more data are required to determine if there is a correlation between size, metabolic rate, flight style and parabronchial morphology.

There are two major traits that can be gleaned from these new models: (i) V3 curves caudally in contrast to the two cranial ventrobronchi and lines nearly the entirety of the ventromedial aspect of the gas-exchanging lung; (ii) the paleopulmonic parabronchi are broken up into large regionalized segments that are divided between the first two dorsobronchi and V1−2, and the caudal dorsobronchi and V3. This suggests that there may be differing functional ventilatory loops in the gas-exchanging lung that may play different roles during both inspiration and expiration. It is possible that these may be tied to ecology and size, and not present in all taxa. While this isolation of parabronchial segments has not been identified in other avian taxa, the large V3 is present in ostriches [27] and African grey parrots [26]. The parrots additionally have a V4 that is substantial and occupies the ventromedial length of the lung along with a large V3, suggesting a similarly important role in maintaining unidirectional airflow through the paleopulmonic parabronchi for both of these bronchi.

### (c) Potential constraints on the inspiratory valving mechanism

A few similar patterns emerge that are shared between zebra finches and African grey parrots: both taxa exhibit reduced intraspecific variability in primary bronchus cross-sectional area (figure 4*a*,*d*) and V2 bronchial ostial areas (figure 4*b*,*e*), the latter being associated with the inspiratory valve. Additionally, both taxa show constrained interostial distances (figure 4*c*,*f*). However, in contrast to our prediction that the entire region of the inspiratory valve would be tightly constrained (i.e. V1−4), this does not appear to be the case, as the area of the ostium of V3 in the zebra finch was seemingly not constrained across all five specimens (figure 4*b*). Interestingly, the primary and secondary bronchial areas for D4 and D5 have lower relative standard error than the more cranially branching dorsobronchi (D1−2) in both taxa. Considering that the mechanism associated with the expiratory valve is poorly understood [79,80], the driving factors underpinning why this may have evolved remains unclear. Both avian species show a diastema or gap between the last ventrobronchus and first dorsobronchus, but this space is more constrained in the zebra finches than the African grey parrots (figure 4*c*,*f*). Our initial hypothesis was that all of the metrics associated with the ventrobronchi would be more constrained than the more caudally positioned secondary airways, but the morphology of V3 (and V4 in the African grey parrots) and separation of the two parabronchial ‘networks’ (figure 3) may be complicating the commonly accepted inspiratory valving hypothesis [46–51].

## 5. Conclusions

The respiratory anatomy of the zebra finch exhibits caudal thoracic air sacs that serve as the primary large sac in the ‘caudal group’ similar to other passerines (e.g. jays, crows, song thrushes, jackdaws, house sparrows, budgerigars) and the violet-eared hummingbird [32,75], but in contrast to the morphology of African grey parrots, ostriches, chickens and ducks [26,27,32]. The use of µCT allowed for visualization of distinct individual parabronchi that connect to specific secondary bronchial segments—an anatomical arrangement that we posit may be coupled with airflow patterns in this species. Considering that this comparison is only between two phylogenetically different taxa, many more species will need to be analyzed to determine if there are broader patterns within the avian bronchial tree that are specifically constrained by the location of the inspiratory and expiratory valves.

## Data Availability

Data is provided as supplementary material. In addition, zebra finch scan data (Morphosource): https://www.morphosource.org/projects/000649397?locale=en. African grey parrot scan data: MorphoSource [81]. African grey parrot measurements: supplemental material, [26]. Supplementary material is available online [82].
